# Prognostic significance of glypican-3 in hepatocellular carcinoma: a meta-analysis

**DOI:** 10.1186/1471-2407-14-104

**Published:** 2014-02-18

**Authors:** Wei-Kai Xiao, Chao-Ying Qi, Dong Chen, Shao-Qiang Li, Shun-Jun Fu, Bao-Gang Peng, Li-Jian Liang

**Affiliations:** 1 Department of Hepatobiliary Surgery, The First Affiliated Hospital, Sun Yat-sen University, No. 58 Zhongshan Er Road, Guangzhou 510080, China; 2 The Operating Center, the First Affiliated Hospital, Sun Yat-sen University, No. 58 Zhongshan Er Road, Guangzhou 510080, China

**Keywords:** Glypican-3, Hepatocellular carcinoma, Prognosis

## Abstract

**Backgrounds:**

Glypican-3(GPC3) has been implicated in tumor development and progression for several years. However, the prognostic significance of GPC3 expression in patients with hepatocellular carcinoma (HCC) is controversial. We performed a meta-analysis of available studies to assess whether GPC3 can be used as a prognostic factor in patients with HCC.

**Methods:**

We searched PubMed and Ovid EBM Reviews databases and evaluated the reference list of relevant articles for studies that assessed the prognostic relevance of GPC3 in patients with HCC. Meta-analysis was performed using hazard ratio (HR) or odds ratio (OR) and 95% confidence intervals (95% CIs) as effect measures.

**Results:**

A meta-analysis of eight studies included 1070 patients was carried out to evaluate the association between GPC3 and overall survival (OS) and disease-free survival (DFS) in HCC patients. The relation between GPC3 and tumor pathological features was also assessed. Our analysis results indicated that high GPC3 expression predicted poor OS (HR: 1.96, 95% CI: 1.51–2.55) and DFS (HR: 1.99, 95% CI: 1.57-2.51) of patients with HCC. GPC3 overexpression was significantly associated with high tumor grade (OR: 3.30, 95% CI: 2.04–5.33), late TNM stage (OR: 2.26, 95% CI: 1.00–5.12), and the presence of vascular invasion (OR: 2.43, 95% CI: 1.23–4.82).

**Conclusions:**

GPC3 overexpression indicates a poor prognosis for patients with HCC, and it may also have predictive potential for HCC invasion and metastasis.

## Background

Hepatocellular carcinoma (HCC) is the sixth most common devastating neoplasm worldwide with increasing incidence over the last several decades across the world [1]. Meanwhile, its third cancer-related mortality rate among varieties of cancers indicates the poor prognosis. Operative resection and liver transplantation are considered potential curative treatments for HCC; however, the overall prognosis of HCC patients remains dismal because of high rate of recurrence after curative resection [2]. Therefore, it is important to identify molecular markers for prognosis of patient survival and/or tumor recurrence, which would help clinician to adopt preventive strategies for patients at high risk of recurrence.

Glypican-3 (GPC3), a member of the glypican family of heparan-sulfate proteoglycans (HSPGs), is bound to the plasma membrane through a glycosyl phosphatidylinositol (GPI) anchor. GPC3 is highly expressed in HCC but not in normal adult tissue. It has been discovered as a good serologic and immunohistochemical diagnostic marker for HCC in recent years [3]. GPC3 would be a more reliable tumor marker that could allow an earlier diagnosis of HCC when compared with serum alpha-fetoprotein [4,5]. Recently, several studies showed that GPC3 can stimulate the growth, migration and adhesion of HCC cells by up-regulating autocrine/paracrine canonical Wnt signaling [6-10]. Moreover, Ho et al identified that GPC3, as one of leading genes, was distinctly expressed in liver CD90^+^ cancer stem cells, which plays an important role in tumor progression and metastasis [11]. Thus, GPC3 expression may function as a new and independent prognostic marker for HCC patients. However, conflicting data have emerged regarding the ability of GPC3 to predict disease recurrence and patients’ outcomes. Therefore, it is necessary to conduct a meta-analysis to systematically and comprehensively understand the prognostic value of GPC3 in HCC.

In this study, we aimed to assess the prognostic significance of GPC3 for OS and DFS in HCC patients by pooling outcomes from the available data. In addition, the correlation between GPC3 expression and tumor pathological features (such as tumor grade, stage, or vascular invasion) was also examined.

## Material and methods

### Identification and selection of studies

#### Study objectives

The primary endpoint was to evaluate patients’ OS and DFS based on their GPC3 profiles. The secondary endpoint was to assess the relation between GPC3 expression and tumor pathological features (such as tumor grade, stage, or vascular invasion, etc.).

#### Search strategy

PubMed and Ovid EBM Reviews databases were systematically searched in August 2013 without time restriction. The search strategy was based on combinations of the following terms: (GPC3 [MESH] or GPC3 [TEXT WORD]) AND (carcinoma, hepatocellular [MESH] or HCC [TEXT WORD]). Reports in English were eligible for inclusion. The reference list was also checked for relevant articles. Investigators were contacted and asked to supply additional data when key information relevant to the meta-analysis was missed.

#### Inclusion criteria of studies

All studies included in this meta-analysis must meet the following criteria: (1) GPC3 expression was measured by immunohistochemistry (IHC); (2) The relationship between GPC3 and DFS and/or OS of patients with HCC was evaluated; (3) Sample size was greater than 20.

#### Definitions and data extraction

OS was defined as the interval between the medical treatment (including liver resection, liver transplantation or radiofrequency ablation, etc.) and the death or the last observation of patients. DFS was measured from the date of treatment to the date of detection of tumorrecurrence. Tumor vascular invasion was defined as presence of either macro- or microscopic vascular invasion. The histologic grade of tumor was assigned according to the Edmondson Steiner grading system, and tumors were grouped as well/moderately (I/II) and poorly (III/IV) degrees of differentiation [12]. The clinical stage of tumors was determined according to the tumor-nodes-metastasis (TNM) classification system of the International Union against Cancer by the American Joint Committee (UICC, 6th edition) [13]. Tumor multifocality was defined as tumor number greater than 2. All data extractions were performed separately by XWK and QCY. Disagreements were resolved by discussion.

#### Qualitative assessment

The quality assessment of included studies was evaluated by the modified Newcastle–Ottawa quality assessment scale with moderate modifications (see the ‘List of Saints’ section) [14,15]. A score of 0-9 (labeled as stars) was used to indicate the quality of each study. Studies labeled with six or more stars were considered to be high quality.

Newcastle-Ottawa quality assessment scale

Selection

(1) Representativeness of the exposed cohort

(a) Truly representative of the average HCC patients in the community*

(b) Somewhat representative of the average HCC patients in the community*

(c) Selected group of users (e.g., nurses, volunteers)

(d) No description of the derivation of the cohort

(2) Selection of the non exposed cohort

(a) Drawn from the same community as the exposed cohort*

(b) Drawn from a different source

(c) No description of the derivation of the non exposed cohort

(3) Ascertainment of exposure (Proof of HCC and glypican-3 measurement)

(a) Secure record (e.g., surgical records)*

(b) Structured interview *

(c) Written self report

(d) No description

(4) Demonstration that outcome of interest was not present at start of study

(a) Yes*

(b) No

Comparability

(1) Comparability of cohorts on the basis of the design or analysis

(a) Study controls for recurrence or metastasis*

(b) Study controls for any additional factor (Age, gender, grade, alpha-fetoprotein level, etc.)*

Outcome

(1) Assessment of outcome

(a) Independent blind assessment*

(b) Record linkage*

(c) Self report

(d) No description

(2) Was follow-up long enough for outcomes to occur? (Death or recurrence)

(a) Yes (3 years)*

(b) No

(3) Adequacy of follow up of cohorts

(a) Complete follow up – all subjects accounted for*

(b) Subjects lost to follow up unlikely to introduce bias – small number lost – (25%) follow up, or description provided of those lost)*

(c) Follow up rate (<75%) and no description of those lost

(d) No statement

A study can be awarded a maximum of one star (*) for each numbered item within the Selection and Outcome categories. A maximum of two stars can be given for Comparability. Underlined and quoted phrases are provided in the scale to allow for adjustment to particular studies. Italicised phrases indicate our interpretation of the question relevant to this study.

### Quantitative analysis (meta-analysis)

#### Statistical methods

Included studies were divided into two groups for analysis: those with data regarding OS and those regarding DFS. Data on the prognostic ability of GPC3 overexpression for OS and DFS were pooled across studies. For the quantitative aggregation of the survival results, hazard ratios (HRs) and their associated standard errors (SEs) were pooled to give the effective value. When these statistical variables were not directly provided in the original articles, they were calculated from available numerical data using methods reported by Parmar et al. [16]. For the pooled analysis of the relation between GPC3 and tumor pathological features, odds ratios (OR) and their 95% confidence intervals (95% CIs) were pooled to give the effective value.

In this study, the cut-off value for "high versus low" GPC3 expression was determined by investigators of each study. A uniform of "high" GPC3 cut-off value was not obtained in this study. A HR >1 implies a worse prognosis in the group with GPC3 overexpression. An OR > 1 indicated higher probability for high tumor grade, later stage or the presence of vascular invasion in the group with GPC3 overexpression. The point estimate of the HR or OR was considered statistically significant at the *p* < 0.05 level if the 95% CI did not include the value 1. In the course of data pooling, we measured the extent of inconsistency among the results by using the I squared (I^2^) statistic and tested the heterogeneity using chi-square (χ^2^) test. Because this test has poor power in the case of few studies, we considered both the presence of significant heterogeneity at the 10% level of significance and the value of I^2^ exceeding 50% as an indicator of significant heterogeneity [17]. The random-effects model was used if there was heterogeneity between studies; otherwise, the fixed-effects model was used [17]. Subgroup analyses that considered more homogeneous studies were performed to identify the best cut-off value of high GPC3 to predict the prognosis of HCC. To determine the extent to which the combined risk estimate might be affected by individual studies, sensitivity analysis was performed by a repetition of the original analysis with the exclusion of the most heavily weighted study. Analysis on main results was performed by using Review Manager Version 5.0 software (Copenhagen: The Nordic Cochrane Centre; The Cochrane Collaboration, 2008).

## Results

### Selection and characteristics of studies

308 records regarding the association of GPC3 and HCC were identified via the initial literature search. 295 studies were excluded after screening the titles or abstracts as they were review articles, abstracts, experiment research, duplicate reports, reports in language other than English or studies irrelevant to the current analysis. 13 relevant studies were selected for detailed evaluation, 4 were further excluded after full assessment due to lacking relevant survival data. After careful evaluation by applying our inclusion criteria, a total of 9 eligible studies were identified [18-26]. Of the 9 studies, 2 were reported by the same study center [22,23], and the patients were partly overlapping in the 2 studies. To avoid duplicate counting, only 1 study with more complete data was selected [23]. Therefore, eight studies with 1070 patients which met our inclusion criteria were selected for our meta-analysis finally [18-21,23-26]. Four studies were performed in China [20,23,25,26], one in Taiwan [21], and three in Japan [18,19,24]. Surgical resection as initial treatment for HCC was reported in 7 studies [18-21,24-26], and liver transplantation(LT) reported in one study [23]. Sample sizes ranged from 31 to 362. Mean or median age ranged from 43 to 65.5 years. The number of male population varied from 29 to 324. The number of HCC patients with vascular invasion ranged from 14 to 107.

DFS was reported or estimated in seven studies [18-21,23-25], whereas OS was only presented in six studies [18-20,24-26]. The scores of study quality assessed by Newcastle-Ottawa quality assessment scale ranged from 5 to 8, with a mean of 6.25. A high score indicated better methodology. HRs were recorded for each study using available data or the methods described above. The number of HCC patients with high GPC3 expression ranged from 20 to 228. The basic features of the eight studies were summarized in Table 1.

**Table 1:** **Baseline characteristics of the studies in the meta**-**analysis**

**Studies (reference)**	**Year**	**Country**	**Treatment**	**Sample size (male, n)**	**Mean/median ages (years)**	**Vascular invasion (yes)**	**Differentiation (I,II/III,IV,n)**
Shirakawal [18]	2009	Japan	SR	107(85)	63.6/60.2	57	12/95
Yorita [19]	2010	Japan	SR	194(142)	NA	108	179/15
Su [20]	2012	China	SR	61(55)	48.0	NA	NA
Yu [21]	2012	Taiwan	SR	100(90)	51.39	23	NA
Wang [23]	2012	China	LT	31(29)	49	16	6/25
Chen [24]	2013	Japan	SR	55(36)	65.5 /63.5	NA	NA
Fu [25]	2013	China	SR	160(140)	50.2	30	123/37
Liang [26]	2013	China	SR	362(324)	43-50	53	NA
**Studies (Reference)**	**Tumor stage (I,II/III,IV,n)**	**Follow-up Mean/median (months)**	**Outcome indexes**	**Multi-variate analysis**	**Patients with High GPC3**	**Study quality (Points)**	**"high" GPC3 cut-off level**
Shirakawal [18]	92/15	42	OS/DFS	Yes	87	8/9	>10 %
Yorita [19]	82/112	31.2	OS/DFS	Yes	97	7/9	≥20 %
Su [20]	45/16	NA	OS/DFS	Yes	32	5/9	>10 %
Yu [21]	NA	66.5	DFS	NA	NA	5/9	+3*
Wang [23]	12/19	24	DFS	NA	20	5/9	>10 %
Chen [24]	NA	66	OS/DFS	yes	28	6/9	>10 %
Fu [25]	NA	34.5	OS/DFS	yes	109	8/9	>25 %
Liang [26]	NA	34.5	OS	NA	228	6/9	>0 %
**Studies (Reference)**	**Antibody type**		**Detection method**		**Evaluation method**	**GPC3 staining**	
Shirakawal [18]	Mouse Monoclonal antibody		IHC		Positive area	Cytoplasm membrane	
Yorita [19]	Monoclonal antibody		IHC: Histofine Her2 kit		Positive area	Cytoplasm membrane	
Su [20]	Mouse Monoclonal antibody		IHC: Chemmate EnVision/Mo&Rb detection kit		Positive area and intensity	Cytoplasm membrane	
Yu [21]	NA		IHC		NA	Cytoplasm membrane	
Wang [23]	Monoclonal antibody		IHC		Positive area	Cytoplasm membrane	
Chen [24]	Rabbit monoclonal antibody		IHC		Positive area	cytoplasm peri- canalicular	
Fu [25]	Mouse Monoclonal antibody		IHC		Percentage of positive cells	Cytoplasm membrane	
Liang [26]	NA		IHC: two-step protocol (DakoCytomation, Glostrup, Denmar)		TMAJ Image application	Cytoplasm membrane	

SR, surgical resection; LT, livertransplantation; NA, not available; IHC, immunohistochemistry; OS, overall survival; DFS, disease-free survival.

Tumor vascular invasion was defined as presence of either macro- or microscopic vascular invasion (including portal vein invasion, etc.).

High glypican-3 cut-off level was scored according to the percentage of positive tumor cells by immunohistochemical staining.

*The expression level was scored on a scale from -3 (underexpression) to +3 (overexpression).

All these eight studies detected GPC3 by IHC. Regarding antibody type, mouse monoclonal antibody was most common use. The immunohistochemical results were evaluated according to the area or percentage of GPC3-positive staining cells in five studies, positive area and expression intensity in one study, and image analysis in one study. The GPC3 expression was mainly in the cytoplasm, with some detected on the cell membranes in all included studies. The cut-off values of high GPC3 expression were 0% [26], 10% [18,20,23,24], 20% [19], and 25% [25] of positive tumor cells by IHC staining reported in the included studies, respectively (Table 1).

### GPC3 expression and OS in HCC patients

**Figure 1:**
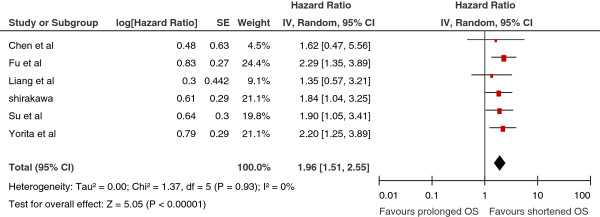
**Meta-analysis of the association between glypican-3(GPC3) overexpression and overall survival (OS) of patients with HCC.** Results are presented as individual and pooled hazard ratio (HR), and 95% confidence interval (CI).

Six studies reported data on GPC3 expression and OS in HCC initially treated by surgical resection [18-20,24-26]. High GPC3 expression was significantly associated with poor OS in all studies. Pooled data from all these studies suggested that high GPC3 expression was significantly correlated with poor OS with a pooled HR estimate of 1.96 (95% CI: 1.51–2.55, *p* = 0.000; Figure 1), without any heterogeneity in the data (χ^2^ = 1.37, I^2^ = 0.0%, *p* = 0.988).

### GPC3 expression and DFS in HCC patients

**Figure 2:**
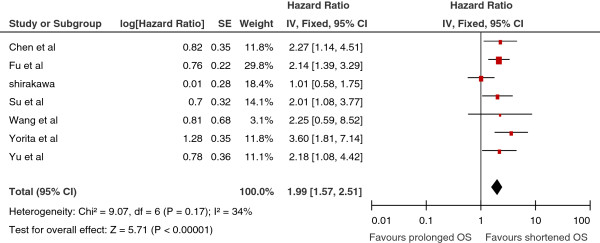
**Meta-analysis of the association between glypican-3(GPC3) overexpression and disease-free survival (DFS) of patients with HCC.** Results are presented as individual and pooled hazard ratio (HR), and 95% confidence interval (CI).

Seven studies reported the relationship between GPC3 expression and DFS in HCC. Six studies [18-21,23-25] presented the information of GPC3 expression correlated with DFS in HCC initially treated by surgical resection. High GPC3 expression was significantly associated with poor DFS in five studies. Pooled data from all seven studies showed that high GPC3 expression was significantly correlated with poor DFS with a pooled estimate HR of 1.99 [95% CI: 1.57–2.51, *p* = 0.000; Figure 2], and without significant heterogeneity in the data (χ^2^ = 9.07, I^2^ = 34%, *p* = 0.17).

### GPC3 expression and tumor pathological features

**Figure 3:**
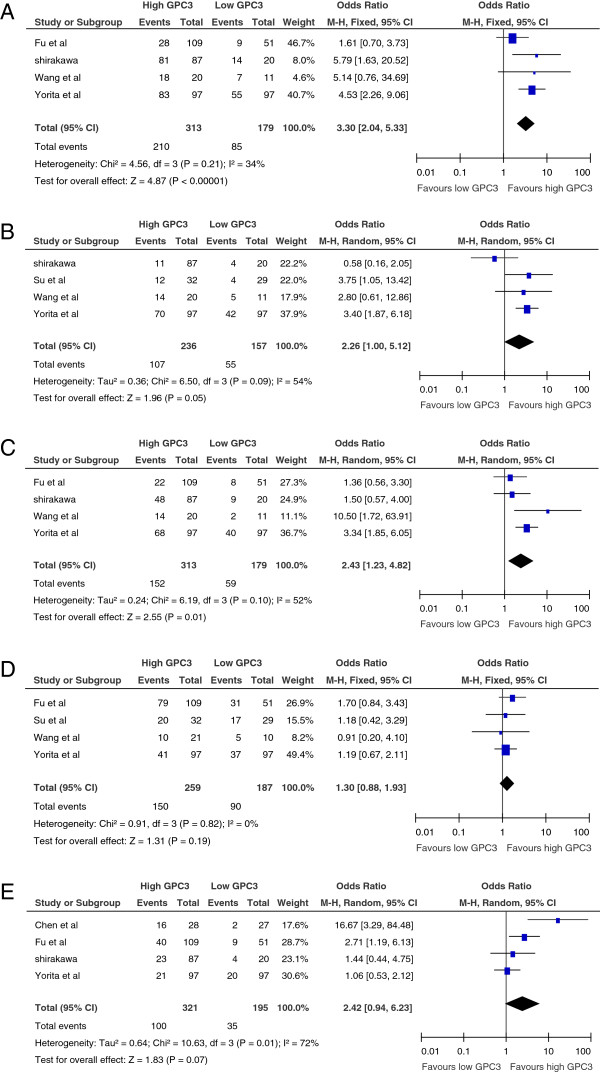
**Meta-analysis of the correlation between glypican-3(GPC3) overexpression and high tumor grade (3A), tumor TNM stage (3B), vascular invasion (3C), tumor size ≥ 5 cm (3D) and tumor multifocality (3E) in HCC.** Results are presented as individual and pooled odds ratio (OR), and 95% confidence interval (CI).

High GPC3 expression tended to be correlated with high tumor grade (moderate and poor differentiation) in four studies [18,19,23,25], and a statistical significant correlation was observed in three studies [18,19,23]. Pooled data from all four studies showed a significant correlation between high GPC3 expression and high tumor grade (OR: 3.30, 95% CI: 2.04–5.33, p = 0.000; Figure 3A).

Four studies also provided data on the correlation of GPC3 with tumor TNM stage [18-20,23]. High GPC3 expression tended to be correlated with late tumor stage (III + IV) in 3 studies [19,20,23], and a statistical significant correlation was observed in two studies [19,20]. Pooled data from all four studies showed a significant correlation between high GPC3 expression and late TNM stage (OR: 2.26, 95% CI: 1.00–5.12, *p* = 0.05; Figure 3B).

Four studies reported data on GPC3 expression and vascular invasion in HCC [18,19,23,25]. High GPC3 expression tended to be correlated with the presence of vascular invasion in all studies, and a statistical significant correlation was observed in two studies [23,25]. Pooled data from all four studies showed a correlation between high GPC3 expression and the presence of vascular invasion (OR: 2.43, 95% CI: 1.23–4.82, *p* = 0.01; Figure 3C).

Four studies reported data on GPC3 expression and tumor size ≥ 5 cm in HCC [19,20,23,25]. High GPC3 expression tended to be correlated with the presence of tumor size ≥ 5 cm in three studies, thought it was not significant difference. Pooled data from all four studies showed high GPC3 expression tended to be correlated with the tumor size ≥ 5 cm (OR: 1.30, 95% CI: 0.88–1.93, *p* = 0.19; Figure 3D).

Four studies reported data on GPC3 expression and tumor multifocality in HCC [18,19,24,25]. High GPC3 expression tended to be correlated with the presence of tumor multifocality in two studies. Pooled data from all four studies showed high GPC3 expression tended to be correlated with the presence of tumor multifocality (OR: 2.42, 95% CI: 0.94–6.23, *p* = 0.07; Figure 3E).

### Subgroup and sensitivity analysis

Subgroup analysis was performed to evaluate whether the pooled estimates of OS, DFS, tumor grade, TNM stage, vascular invasion, tumor size ≥ 5 cm and tumor multifocality were different according to the GPC3 cut-off values of 0%, 10%, 20%, and 25% of positive tumor cells by IHC staining reported in the included studies. In addition, subgroup analysis of studies that only used multivariate statistical analysis was also conducted (Table 2). Sensitivity analyses were performed by exclusion of the highest weighted study in each pooled analysis (Table 3). Finally, the results were all consistent with the above outcomes.

**Table 2:** **Multivatiate analysis for disease**-**free and overall survival in our included studies**

**Study (Reference)**	**Multivariate analysis**	**DFS**			**OS**		
		**HR**	**95% CI**	***P***	**HR**	**95% CI**	***P***
Shirakawal^18^	Yes	NA	NA	NA	1.84	1.04-3.25	0.034
Yorita^19^	Yes	3.6	1.8-7.1	0.0003	1.9	0.7-5.1	0.176
Su^20^	Yes	NA	NA	NA	1.905	1.063-3.415	0.030
Yu^21^	NA	NA	NA	NA	NA	NA	NA
Wang^23^	NA	NA	NA	NA	NA	NA	NA
Chen^24^	Yes	2.27	1.14-4.51	0.02	NA	NA	NA
Fu^25^	Yes	2.14	1.39-3.29	0.001	2.29	1.35-3.89	0.002
Liang^26^	NA	NA	NA	NA	NA	NA	NA

NA, not available; OS, overall survival; DFS, disease-free survival. Hazard ratio (HR); Odds ratio (OR); confidence intervals (CIs).

**Table 3:** Subgroup and Sensitivity analysis

Variables (cut-off value)*	**Number of study**	**HR/OR**	**95% CI**	**p**	**I**^**2**^
**OS**	6	1.96	1.51-2.55	0.000	0%
**0% cut-off value Of GPC3**	1	1.35	0.57-3.21	0.000	NA
**10% cut-off value Of GPC3**	3	1.84	1.25-2.71	0.002	0%
**20% cut-off value Of GPC3**	1	2.20	1.25-3.89	0.006	NA
**25% cut-off value Of GPC3**	1	2.29	1.35-3.89	0.002	NA
**Exclusion of Liang et al.**	5	2.04	1.55-2.68	0.000	0%
Studies with multivariate analysis	4	2.06	1.56-2.73	0.000	0%
Studies without multivariate analysis	2	1.43	0.70-2.91	0.32	0%
**DFS**	7	1.99	1.57-2.51	0.000	34%
**0% cut-off value Of GPC3**	0	NA	NA	NA	NA
**10% cut-off value Of GPC3**	4	1.60	1.14-2.25	0.007	33%
**20% cut-off value Of GPC3**	1	3.60	1.81-7.14	0.000	NA
**25% cut-off value Of GPC3**	1	2.14	1.39-3.29	0.000	NA
**Exclusion of Yorita et al.**	6	1.83	1.43-2.36	0.000	14%
Studies with multivariate analyis	3	2.43	1.76-3.35	0.000	0%
Studies without multivariate analyis	4	1.58	1.12-2.22	0.010	29%
**Tumor grade**	4	3.30	2.04-5.33	0.000	34%
**0% cut-off value Of GPC3**	0	NA	NA	NA	NA
**10% cut-off value Of GPC3**	2	5.55	1.92-16.06	0.002	0%
**20% cut-off value Of GPC3**	1	4.53	2.26-9.06	0.000	NA
**25% cut-off value Of GPC3**	1	1.61	0.70-3.73	0.26	NA
**Exclusion of Yorita et al. l**	3	2.45	1.26-4.78	0.008	39%
**TNM stage**	4	2.26	1.00-5.12	0.05	54%
**0% cut-off value Of GPC3**	0	NA	NA	NA	NA
**10% cut-off value Of GPC3**	3	1.78	0.54-5.87	0.34	57%
**20% cut-off value Of GPC3**	1	3.40	1.87-6.18	0.000	NA
**25% cut-off value Of GPC3**	0	NA	NA	NA	NA
**Exclusion of Yorita et al.**	3	1.78	0.54-5.87	0.34	57%
**Vascular invasion**	4	2.43	1.23-4.82	0.01	52%
**0% cut-off value Of GPC3**	0	NA	NA	NA	NA
**10% cut-off value Of GPC3**	2	3.41	0.52-22.41	0.20	71%
**20% cut-off value Of GPC3**	1	3.34	1.85-6.05	0.000	NA
**25% cut-off value Of GPC3**	1	1.36	0.56-3.30	0.50	NA
**Exclusion of Yorita et al.**	3	2.11	0.81-5.49	0.13	52%
**Tumor size** ≧ **5 cm**	4	1.30	0.88-1.93	0.19	0%
**0% cut-off value Of GPC3**	0	NA	NA	NA	NA
**10% cut-off value Of GPC3**	2	1.08	0.46-2.53	0.78	0%
**20% cut-off value Of GPC3**	1	1.19	0.67-2.11	0.56	NA
**25% cut-off value Of GPC3**	1	1.70	0.84-3.43	0.14	NA
**Exclusion of Yorita et al.**	3	1.41	0.82-2.42	0.21	0%
**Tumor multifocality**	4	2.42	0.94-6.23	0.07	72%
**0% cut-off value Of GPC3**	0	NA	NA	NA	NA
**10% cut-off value Of GPC3**	2	4.59	0.41-51.05	0.21	83%
**20% cut-off value Of GPC3**	1	1.06	0.53-2.12	0.86	NA
**25% cut-off value Of GPC3**	1	2.71	1.19-6.13	0.02	NA
**Exclusion of Yorita et al.**	3	3.50	1.09-11.18	0.03	66%

GPC3:glypican3; HR: hazard ratio; OR: odds ratios; CI: confidence interval; OS: overall survival; DFS: disease-free survival; NA: not available.

*High glypican-3 cut-off level was scored according to 0%, 10%, 20%, and 25% of the percentage of positive tumor cells by immunohistochemical staining.

A HR >1 implies a worse prognosis in the group with GPC3 overexpression; while an OR > 1 indicated higher probability for high tumor grade, later TNM stage, the presence of vascular invasion,tumor size ≥ 5 cm and tumor multifocality in HCC with GPC3 overexpression.

### Publication bias

Publication bias estimate was mainly used to evaluate the reliability of meta-analysis results, especially for those showing statistical significance [27]. Publication bias was assessed by using Egger’s test [28]. Statistical significance was set at *p* <0.10. The results suggested evidence for publication bias in OS studies (p = 0.001), while it did not reveal significant publication bias in DFS studies (p = 0.404).

## Discussion

HCC, one of the most aggressive malignant tumors, is associated with poor prognosis despite significant improvement in surgical and locoregional therapies in past few decades [20]. In recent years, considerable interest has been focused on the mechanism of HCC metastasis/recurrence after operation. However, the molecular factors associated with tumor progression and invasion of HCC are still unknown.

From a clinical perspective, therefore, it is of great significance to identify the most useful biomarkers for prognostic prediction, which can help clinicians to adopt preventive strategies for patients at high risk and further improve outcome of patient with HCC. One potential candidate marker for the progression and prognosis of HCC is GPC3. Although the association of GPC3 with HCC has been explored for several years, the available data have not yet been fully analyzed. Considering that meta-analysis is a valuable tool in biomarker validation [15], here we conducted a meta-analysis to investigate the association between GPC3 and HCC progression and prognosis.

In this meta-analysis, we first assessed the association of high GPC3 expression with OS and DFS in HCC patients. The pooled outcomes demonstrated that high GPC3 expression significantly predicted poor OS and DFS for patients with HCC (*p* = 0.000, *p* =0.000, respectively). Although the results of the analysis are positive, the reasons for different OS and DFS in HCC patients with high or low GPC3 expression are not yet clear. It should be noted that the poor prognosis of HCC was usually correlated with tumor invasion and metastasis [29]. Several studies have demonstrated that the GPC3 protein regulates HCC cells proliferation and growth, as well as invasion via manipulating the canonical Wnt signaling pathway, fibroblast growth factors, bone morphogenic proteins, transforming growth factor-β, and insulin like growth factor-2 signaling pathways [30-32]. Ruan et al. [33] reported that inhibition of glypican-3 expression via RNA interference influences the growth and invasive ability of the MHCC97-H human HCC cell line. These may, in part, explain the aggressive malignant feature of HCC modulated by GPC3.

Taking into account the association between tumor invasion and GPC3 expression profile, in the present study, we also carried out pooled analyses of the association between GPC3 expression and tumor pathological features. The results indicated that high expression of GPC3 was closely correlated with high tumor grade, late TNM stage and the presence of vascular invasion. In summary, our pooled outcomes supported the hyphothesis that GPC3 overexpression might promote invasion and metastasis of HCC by direct or indirect mechanisms, which subsequently leaded to poor prognosis of HCC.

Our results should be interpreted cautiously since some limitations exist in this present meta-analysis. First, the potential risk of bias was a concern, since the positive results are more likely to be published than negative ones. Though we tried to identify all relevant information, some missing data are still inevitable, as reflecting by our publication bias evaluation. Moreover, total number of included studies, as well as the total sample size was relatively small, which might influence the validity of our analysis to some extent. Second, all these eight studies are based on Asian population. It is believed that distinct site difference exists in HCC between Western and Eastern populations. In Asia, the majority HCC is hepatitis B (Chinese population) or C (Japanese population) virus-related HCC whereas alcohol-related or hepatitis C virus-related HCC is the predominant cancer type in Western countries. Therefore, the biologic features and behaviors of tumors might be different between the two categories. As a result, whether the GPC3 expression status and its function in Western patients are identical with Asian ones is still unknown, because there is no data about Western populations available till now. Third, in this study, GPC3 expression profiles were from tissue-based studies. In view of that serum GPC3 level was an indirect marker of its tissue expression, and it can be easily obtained and measured at any time point. Thus, whether serum-based GPC3 levels were superior to tissue-based GPC3 expression in predicting HCC invasion and prognosis remained to be investigated by further studies. Fourth, the cut-off value for defining high GPC3 expression has not been unified in these studies, which may lead to between-study heterogeneity. Therefore, future large sample study to give a definitive cut-off value of high GPC3 expression with good sensitivity and specificity is needed. Fifth, since our meta-analysis was carried out on the pooled data, strong recommendations at an individual patient level could not be obtained. Sixth, Several factors related to IHC such as the type of antibody use, detection method, evaluation method of results, inter-observer variation lead to the heterogeneity of IHC studies.

However, we addressed the issue of heterogeneity by a rigorous methodologic approach. We included only studies with a minimum sample size of 20 patients and required at least 3 studies to carry out pooled analysis. Furthermore, we also performed subgroup analysis and sensitivity analysis to evaluate potential sources of bias and the observed inter-study heterogeneity. Although we were unable to carried out analysis with regard to certain potential relevant factors (such as patients’ body mass index) due to the lack of data reported in many of the included studies, the key finding of high GPC3 expression in the HCC tissue representing an indicator of poor prognosis in patients with HCC was consistently present in the pooled analysis of all studies as well as throughout all subgroup analysis.

## Conclusions

In summary, current available evidence supports the notion of a strong prognostic effect of high GPC3 expression in patients with HCC. It may be speculated that these patients may potentially benefit from adjuvant therapy. Further studies are required to verify the prognostic significance of serum-based GPC3 levels, as a simple method to monitor response to systemic therapy, tumor progression and prognosis. Moreover, a definitive cut-off value of high GPC3 expression based on future large sample study is recommended.

## Abbreviations

GPC3: Glypican-3; HCC: Hepatocellular carcinoma; OS: Overall survival; DFS: Disease-free survival; HR: Hazard ratio; OR: Odds ratio; CIs: Confidence intervals; IHC: Immunohistochemistry; LT: Liver transplantation; TNM: Tumor-nodes-metastasis.

## Competing interests

The authors declare that they have no conflict of interest.

## Authors’ contributions

LSQ conceived and designed the review, supervised the data collection, statistical analysis and critically revised the manuscript. XWK and QCY carried out the literature search, performed data extraction and data analysis, and wrote the manuscript. CD, FSJ, PBG and LLJ participated in data extraction, and resolved the disagreement. All authors read and approved the final manuscript.

## Pre-publication history

The pre-publication history for this paper can be accessed here:

http://www.biomedcentral.com/1471-2407/14/104/prepub

## References

[B1] Jemal A Bray F Center MM Ferlay J Ward E Forman D Global cancer statistics CA Cancer J Clin 2011 61 69 90 10.3322/caac.20107 21296855

[B2] Tang ZY Ye SL Liu YK Qin LX Sun HC Ye QH Wang L Zhou J Qiu SJ Li Y Ji XN Liu H Xia JL Wu ZQ Fan J Ma ZC Zhou XD Lin ZY Liu KD A decade’s studies on metastasis of hepatocellular carcinoma J Cancer Res Clin Oncol 2004 130 187 96 10.1007/s00432-003-0511-1 14685850 PMC12161827

[B3] Filmus J Selleck SB Glypicans: proteoglycans with a surprise J Clin Invest 2001 108 497 501 10.1172/JCI200113712 11518720 PMC209407

[B4] Llovet JM Chen Y Wurmbach E Roayaie S Fiel MI Schwartz M Thung SN Khitrov G Zhang W Villanueva A Battiston C Mazzaferro V Bruix J Waxman S Friedman SL A molecular signature to discriminate dysplastic nodules from early hepatocellular carcinoma in HCV cirrhosis Gastroenterology 2006 131 1758 1767 10.1053/j.gastro.2006.09.014 17087938

[B5] Wang XY Degos F Dubois S Tessiore S Allegretta M Guttmann RD Jothy S Belghiti J Bedossa P Paradis V Glypican-3 expression in hepatocellular tumors: diagnostic value for preneoplastic lesions and hepatocellular carcinomas. Hum Pathol 2006 37 1435 1441 10.1016/j.humpath.2006.05.016 16949914

[B6] Capurro MI Xiang YY Lobe C Filmus J Glypican-3 promotes the growth of hepatocellular carcinoma by stimulatingcanonical Wnt signaling Cancer Res 2005 65 6245 6254 10.1158/0008-5472.CAN-04-4244 16024626

[B7] Nakatsura T Yoshitake Y Senju S Monji M Komori H Motomura Y Hosaka S Beppu T Ishiko T Kamohara H Ashihara H Katagiri T Furukawa Y Fujiyama S Ogawa M Nakamura Y Nishimura Y Glypican-3, overexpressed specifically in human hepatocellular carcinoma, is a novel tumor marker Biochem Biophys Res Commun 2003 306 16 25 10.1016/S0006-291X(03)00908-2 12788060

[B8] Song HH Shi W Xiang YY Filmus J The loss of glypican-3 induces alterations in Wnt signaling J Biol Chem 2005 280 2116 2125 15537637 10.1074/jbc.M410090200

[B9] Stigliano I Puricelli L Filmus J Sogayar MC Bal De Kier Joffé E Peters M Glypican-3 regulates migration, adhesion and actin cytoskeleton organization in mammary tumor cells through Wnt signaling modulation Breast Cancer Res Treat 2009 114 251 262 10.1007/s10549-008-0009-2 18404367

[B10] Torisu Y Watanabe A Nonaka A Midorikawa Y Makuuchi M Shimamura T Sugimura H Niida A Akiyama T Iwanari H Kodama T Zeniya M Aburatani H Human homolog of NOTUM, overexpressed in hepatocellular carcinoma, is regulated transcriptionally by beta-catenin/TCF Cancer Sci 2008 99 1139 1146 10.1111/j.1349-7006.2008.00814.x 18429952 PMC11158900

[B11] Ho DW Yang ZF Yi K Lam CT Ng MN Yu WC Lau J Wan T Wang X Yan Z Liu H Zhang Y Fan ST Gene expression profiling of liver cancer stem cells by RNA-sequencing PLoS One 2012 7 e37159 10.1371/journal.pone.0037159 22606345 PMC3351419

[B12] Edmondson HA Steiner PE Primary carcinoma of the liver: a study of 100 cases among 48,900 necropsies Cancer 1954 7 462 503 10.1002/1097-0142(195405)7:3<462::AID-CNCR2820070308>3.0.CO;2-E 13160935

[B13] Varotti G Ramacciato G Ercolani G Grazi GL Vetrone G Cescon M Del Gaudio M Ravaioli M Ziparo V Lauro A Pinna A Comparison between the fifth and sixth editions of the AJCC/UICC TNM staging systems for hepatocellular carcinoma: multicentric study on 393 cirrhotic resected patients Eur J Surg Oncol 2005 31 760 767 10.1016/j.ejso.2005.04.008 15975760

[B14] Cota GF De Sousa MR Fereguetti TO Rabello A The Newcastle-Ottawa Scale (NOS) for assessing the quality if nonrandomized studies in meta-analyses 2013 http://www.ohri.ca/programs/clinical_epidemiology/oxford.asp

[B15] Zhang CH Xu GL Jia WD Ge YS Li JS Ma JL Ren WH Zhang CH Xu GL Jia WD Prognostic significance of osteopontin in hepatocellular carcinoma: a meta-analysis Int J Cancer 2012 130 2685 2692 10.1002/ijc.26301 21780114

[B16] Parmar MK Torri V Stewart L Extracting summary statistics to perform metaanalyses of the published literature for survival endpoints Stat Med 1998 17 2815 3284 10.1002/(SICI)1097-0258(19981230)17:24<2815::AID-SIM110>3.0.CO;2-8 9921604

[B17] Higgins J Green S Cochrane handbook for systematic reviews of interventions 2008 New York, NY: Cochrane Collaboration, John Wiley and Sons

[B18] Shirakawa H Suzuki H Shimomura M Kojima M Gotohda N Takahashi S Nakagohri T Konishi M Kobayashi N Kinoshita T Nakatsura T Glypican-3 expression is correlated with poor prognosis in hepatocellular carcinoma Cancer Sci 2009 100 1403 1407 10.1111/j.1349-7006.2009.01206.x 19496787 PMC11158276

[B19] Yorita K Takahashi N Takai H Kato A Suzuki M Ishiguro T Ohtomo T Nagaike K Kondo K Chijiiwa K Kataoka H Prognostic significance of circumferential cell surface immunoreactivity of glypican-3 in hepatocellular carcinoma Liver Int 2011 31 120 131 10.1111/j.1478-3231.2010.02359.x 20964802

[B20] Su N Chen B Huang N Shen P Ding Y Ye XH Zeng FY Zheng DY Luo RC Glypican-3, a novel prognostic marker of hepatocellular cancer, is related with postoperative metastasis and recurrence in hepatocellular cancer patients Mol Biol Rep 2012 39 351 357 10.1007/s11033-011-0745-y 21655958

[B21] Yu MC Lee YS Lin SE Wu HY Chen TC Lee WC Chen MF Tsai CN Recurrence and poor prognosis following resection of small hepatitis B-related hepatocellular carcinoma lesions are associated with aberrant tumor expression profiles of glypican 3 and osteopontin Ann Surg Oncol 2012 19 Suppl3 455 463 21822558 10.1245/s10434-011-1946-2

[B22] Wang Y Shen Z Zhu Z Han R Huai M Clinical values of AFP, GPC3 mRNA in peripheral blood for prediction of hepatocellular carcinoma recurrence following OLT: AFP, GPC3 mRNA for prediction of HCC Hepat Mon 2011 11 195 9 22087143 PMC3206678

[B23] Wang YL Zhu ZJ Teng DH Yao Z Gao W Shen ZY Glypican-3 expression and its relationship with recurrence of HCC after liver transplantation World J Gastroenterol 2012 18 2408 2414 10.3748/wjg.v18.i19.2408 22654434 PMC3353377

[B24] Chen IP Ariizumi SI Nakano M Yamamoto M Positive glypican-3 expression in early hepatocellular carcinoma predicts recurrence after hepatectomy J Gastroenterol 2013 in press 10.1007/s00535-013-0793-2 PMC3895193 23532638

[B25] Fu SJ Qi CY Xiao WK Li SQ Peng BG Liang LJ Glypican-3 is a potential prognostic biomarker for hepatocellular carcinoma after curative resection Surgery 2013 154 536 544 10.1016/j.surg.2013.02.014 23601901

[B26] Liang J Ding T Guo ZW Yu XJ Hu YZ Zheng L Xu J Expression pattern of tumour-associated antigens in hepatocellular carcinoma: association with immune infiltration and disease progression Br J Cancer 2013 109 1031 1039 10.1038/bjc.2013.390 23868000 PMC3749565

[B27] E Y He N Wang Y Percutaneous transluminal angioplasty (PTA) alone versus PTA with balloonexpandable stent placement for short-segment femoropopliteal artery disease: a meta-analysis of randomized trials J Vasc Interv Radiol 2008 19 499 503 10.1016/j.jvir.2007.12.446 18375292

[B28] Egger M Davey Smith G Schneider M Minder C Bias in meta-analysis detected by a simple, graphical test BMJ 1997 315 629 634 10.1136/bmj.315.7109.629 9310563 PMC2127453

[B29] Tung-Ping Poon R Fan ST Wong J Risk factors, prevention, and management of postoperative recurrence after resection of hepatocellular carcinoma Ann Surg 2000 232 10 24 10.1097/00000658-200007000-00003 10862190 PMC1421103

[B30] Cheng W Tseng CJ Lin TT Cheng I Pan HW Hsu HC Lee YM Glypican-3-mediated oncogenesis involves the Insulin-like growth factor-signaling pathway Carcinogenesis 2008 29 1319 1326 10.1093/carcin/bgn091 18413366 PMC2500215

[B31] Lai JP Oseini AM Moser CD Yu C Elsawa SF Hu C Nakamura I Han T Aderca I Isomoto H Garrity-Park MM Shire AM Li J Sanderson SO Adjei AA Fernandez-Zapico ME Roberts LR The oncogenic effect of sulfatase 2 in human hepatocellular carcinoma is mediated in part by glypican 3-dependent Wnt activation Hepatology 2010 52 1680 1689 10.1002/hep.23848 20725905 PMC2967616

[B32] Midorikawa Y Ishikawa S Iwanari H Imamura T Sakamoto H Miyazono K Kodama T Makuuchi M Aburatani H Glypican-3, overexpressed in hepatocellular carcinoma, modulates FGF2 and BMP-7 signaling Int J Cancer 2003 103 455 465 10.1002/ijc.10856 12478660

[B33] Ruan J Liu F Chen X Zhao P Su N Xie G Chen J Zheng D Luo R Inhibition of glypican-3 expression via RNA interference influences the growth and invasive ability of the MHCC97-H human hepatocellular carcinoma cell line Int J Mol 2011 28 497 503 10.3892/ijmm.2011.704 21617840

